# Draft-genome sequence of *Shewanella algae* strain C6G3

**DOI:** 10.1186/s40793-015-0022-0

**Published:** 2015-07-23

**Authors:** Axel Aigle, Valerie Michotey, Patricia Bonin

**Affiliations:** Aix Marseille Université, CNRS, Université de Toulon, IRD, MIO UM 110, 13288 Marseille, France

**Keywords:** *Shewanella*, *Shewanella algae*, *Shewanella oneidensis*, Nitrate reduction, Dissimilative reduction of nitrite into ammonium, Metal-oxide reduction

## Abstract

*Shewanella algae* strain C6G3, isolated from the 2 uppermost centimeters of muddy sediment of Arcachon Bay (SW Atlantic French coast, sampled in October 2007) has the capability to use a large panel of terminal electron acceptors under anaerobic condition, such as nitrate, nitrite and metal-oxide, and presents a great metabolic versatility. Here, we present the non-contiguous draft-genome sequence of *Shewanella algae* C6G3, which consists of a 4,879,425 bp. The chromosome contains 5792 predicted genes. In total, the genome consists of 24 rRNA genes, 86 tRNA genes and 5660 genes assigned as protein-coding genes.

**Electronic supplementary material:**

The online version of this article (doi:10.1186/s40793-015-0022-0) contains supplementary material, which is available to authorized users.

## Introduction

The genus *Shewanella* comprises several Gram-negative species which are widely distributed in marine and freshwater environments. *Shewanella algae* (formerly classified as *S. putrefaciens**)* has been frequently isolated from marine water samples and spoiling fish [1–3]. They are capable of reducing trimethylamine N-oxide (TMAO) to trimethylamine and producing hydrogen sulfide, both of which are main components of the fishy odor present during low temperature storage. They were also isolated from human feces, skin and other clinical samples [4, 5]. The collected strains were heterogeneous with G + C values ranging from 43 % to 55 %. However, there were differences between environmental and clinical isolates. Most of the strains isolated from human clinical specimens and identified as *S. putrefaciens* showed beta-hemolysis on sheep blood agar whereas environmental strains were nonhemolytic [6, 7]. During a screening study of heterotrophic bacteria from the sediment of Arcachon Bay [8], a large set of isolates was obtained from different sampling sites and years (2). Among the 24 isolates, 15 strains belong to *Shewanella* genus and were able to reduce Mn(III/IV) and/or nitrate. The genus seems to play an important role in the turnover of organic matter coupled to anaerobic respiration electron acceptors, such as Fe(III), Mn(III/IV) and NO_2/3_. Here we report on further taxonomic and physiological studies on strain *Shewanella algae* strain C6G3 and present its main genomic features.

### Organism information

#### Classification and features

**Fig. 1:**
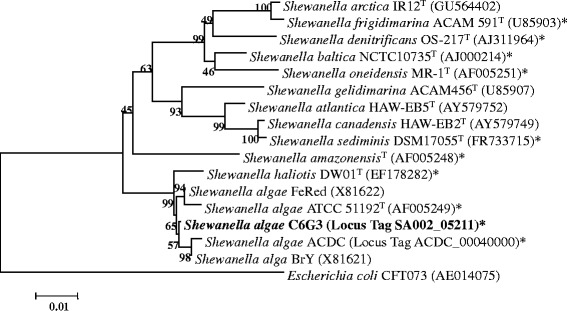
Phylogenetic position of *Shewanella algae* C6G3 relative to the genus *Shewanella* and other strains of *Shewanella algae*. This Neighbor-joining tree is based on 1243 aligned characters of the 16S rRNA gene. The bootstrap percentages higher than 50 % are indicated at the node after 1000 resampled data sets. Branch length corresponds to sequence differences as indicated on the scale bar (substitutions per position). The proposed *Shewanella* species have been chosen from the List of Prokaryotic names with Standing in Nomenclature (type strain and sequence accession number) for their ability to use nitrate, nitrite and / or metal oxides. Species whose genome has been sequenced are marked with star (*). *Escherichia coli* [44] was used as out-group

**Fig. 2:**
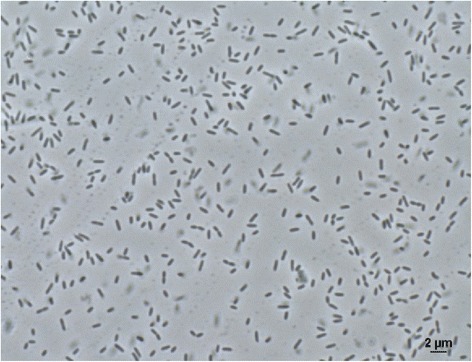
Phase contrast micrograph of *Shewanella algae* C6G3. Bar scale: 2 μm

The genus *Shewanella* currently contains 62 species [9] including *Shewanella algae* ATCC 51192^T^, the first described [2]. Ribosomal gene of strain C6G3 exhibits 99 % similarity with available ribosomal gene of *Shewanella algae* (strains ATCC 51192^T^, ACDC [3], BrY [10] and FeRed [11]) and was affiliated to this specie (Fig. 1). Cells of strain C6G3 are straights rods (Fig. 2), Gram-negative, motile, free-living and non-sporulating. Different growth temperatures, pH and % NaCl were tested (Table 1). Optimal growth occurs at 30 °C, pH 8 and 10 % NaCl (w/v). For strain C6G3, ATCC 51192^T^ and *S. oneidensis* MR-1^T^ [12], the use of 95 carbon sources was tested with Biolog GN2 microplate™ (Microlog) (Additional file 1: Table S1). Strain C6G3 presents 32 positive results: 5/30 carbohydrates, 14/29 organic acids, 8/19 amino-acids. Similar results were obtained for strain ATCC 511392^T^ (38/95). Among the two strains of *S. algae*, slightly different patterns of carbon source were noticed; however, profil of *S oneidensis* MR-1^T^ was different (16/95). Some electron acceptors were also tested for strain C6G3 according to genome annotation and *Shewanella algae* literature [1] (Additional file 1: Table S1).

**Table 1:** Classification and general features of *S. algae* C6G3 [17]

MIGS ID	Property	Term	Evidence code^a^
	Classification	Domain *Bacteria*	TAS [45–47]
		Phylum *Proteobacteria*	TAS [48]
		Class *Gammaproteobacteria*	TAS [49, 50]
		Order *Alteromonadales*	TAS [51]
		Family *Shewanellaceae*	TAS [52]
		Genus *Shewanella*	TAS [53, 54]
		Species *Shewanella algae*	TAS [2]
		(Type) strain: C6G3	IDA
	Gram stain	Negative	IDA
	Cell shape	Straights rods	IDA
	Motility	Motile	IDA
	Sporulation	Nonsporulating	NAS
	Temperature range	10–40 °C (die at 45 °C)	IDA
	Optimum temperature	30 °C	IDA
	pH range; Optimum	6–9; 8	IDA
	Carbon source	Disaccharides, some organic acids, amino acids	IDA
MIGS-6	Habitat	Muddy interdidal sediments	IDA
MIGS-6.3	Salinity	0-10 % NaCl (w/v); 10 % NaCl (w/v)	IDA
MIGS-22	Oxygen requirement	Facultative anaerobic	IDA
MIGS-15	Biotic relationship	Free-living	IDA
MIGS-14	Pathogenicity	Biosafety level 1 for ATCC 51192	TAS [2]
MIGS-4	Geographic location	Arcachon Bay, Aquitaine, France	IDA
MIGS-5	Sample collection	October, 2007	IDA
MIGS-4.1	Latitude	N44° 40’	IDA
MIGS-4.2	Longitude	W1° 10’	IDA
MIGS-4.3	Depth	Top 10 cm of sediment	IDA
MIGS-4.4	Altitude	Sea level	IDA

^a^Evidence codes - IDA: Inferred from Direct Assay; TAS: Traceable Author Statement (i.e., a direct report exists in the literature); NAS: Non-traceable Author Statement (i.e., not directly observed for the living, isolated sample, but based on a generally accepted property for the species, or anecdotal evidence). These evidence codes are from the Gene Ontology project [55]. You will find the table of associated MIGS Record in additional file (Additional file 2)

### Chemotaxonomic data

The fatty acid analysis was performed on two strains of *S. algae* (C6G3, ATCC 51192^T^) and on *S. oneidensis* MR-1^T^ (Additional file 1: Table S2). At the end of aerobic culture, fatty acids were extracted from cell pellet by alkaline hydrolysis and analyzed using chromatography-electron ionization mass spectrometry (GC-EIMS) following the protocol described in Zabeti et al., [13]. The overall fatty acid pattern of *S. algae* C6G3 is rather common for the genus *Shewanella**.* The major ones were C16:1ω7 (35.2 %), C16:0 (34.6 %) (generally reported between 16–55 % and 5–31 %, respectively [14]) and 3OH-C12:0 (7.7 %). Interestingly, the C15:0 br is much lower in strain C6G3 (2.3 %) than in *S. algae* ATCC 51192^T^ and *S. oneidensis* MR-1^T^ (27.4 % and 20.4 %, respectively). *S. algae* C6G3 presents also a relatively higher percentage of short-chain fatty acids (shorter than C15, 16.0 %) than both *S. oneidensis* MR-1^T^ and *S. algae* ATCC 51192^T^ (8.5 %).

### Genome sequencing information

#### Genome project history

*S. algae* C6G3 was isolated from intertidal marine sediment on the basis of its ability to use large range of electron acceptors particularly nitrate, nitrite and metal-oxides [8]. The genome of *S. algae* C6G3 is the second to be reported from that species, the other one being *S. algae* ACDC [15]. The genome project of *S. algae* C6G3 is deposited in the Genome On Line Database [16]. A summary of the project and information on compliance with MIGS version 2.0 [17] are shown (Table 2).

**Table 2:** Genome project information

MIGS ID	Property	Term
MIGS 31	Finishing quality	Non-contiguous finished
MIGS-28	Libraries used	Fragments (mean size 200 pb)
MIGS 29	Sequencing platforms	Semiconductor Ion Torrent PGM
MIGS 31.2	Fold coverage	50×
MIGS 30	Assemblers	SeqMan NGen® (DNASTAR)
MIGS 32	Gene calling method	GLIMMER2 (RAST), GeneMark (v.2.6.r), GenePRIMP (IMG DOE-JGI)
	Locus Tag	fig|22.6.peg. (RAST), SA002_ (IMG DOE-JGI)
	Genbank ID	JPMA00000000 (JPMA01000001-JPMA01000043)
	GenBank Date of Release	March 19, 2015
	GOLD ID	Gi0073428
	BIOPROJECT	PRJNA255462
MIGS 13	Source Material Identifier	SAMN02921234
	Project relevance	Environment

### Growth conditions and DNA isolation

*S. algae* C6G3 was grown aerobically at 30 °C under stirring condition on artificial sea water [18] amended with lactate (3 g/L), yeast extract (1 g/L) and tryptone (5 g/L). DNA was extracted from cells collected in exponential growth phase using the protocol of Marteinsson et al. [19]. DNA concentration and purity were checking on biophotometer® (Eppendorf) before sequencing.

### Genome sequencing and assembly

The genome sequencing of *S. algae* C6G3 was generated at the Molecular Research LP MR DNA Laboratory (USA). De novo whole-genome shotgun sequencing was performed using the Ion Torrent PGM (Life Technologies [20]) sequencing platform. This produced 1,444,981 reads with an average length of 200 bp for a total number of sequenced bases of 288,996,200 representing a sequencing depth of 50-fold. The assembly of *S. algae* C6G3 genome was generated at MR DNA Laboratory using the SeqMan NGen® software assembler (DNASTAR). The final assembly identified 43 contigs generating a genome size of 4,9 Mb.

### Genome annotation

Genome annotation was performed on two platforms: on RAST [21] and on IMG/ER [22] (DOE Joint Genome Institute [23]). The tRNAscan-SE tool [24] (RAST and IMG/ER) was used to find tRNA genes, whereas ribosomal RNAs were detected using RMAmmer [25] (IMG/ER) and tool “search_for_rnas” (developed by Niels Larsen (available by the author), RAST). Open Reading Frames (ORFs) were predicted using GLIMMER2 [26] in RAST and using GeneMark (v.2.6.r) [27] and GenePRIMP [28] as a part of the DOE-JGI genome annotation pipeline. Gene prediction analyses and functional annotations were performed in RAST with a series of BLAST against FIG hands-curated subsystems [29]. They were also analyzed with FIGfams collection databases, and through comparative approaches with Integrated Microbial Genome – Expert Review platform (RPS-BLAST, BLAST, BLASTp, Hmmsearch (HMMER)) against non-redundant databases including COGs, Pfam [30], TIGR-fam [31], KEEG [32], IMG. Additional functional annotations were performed within the SEED framework (RAST) [33] and the IMG/ER (DOE-JGI) platform.

### Genome properties

**Fig. 3:**
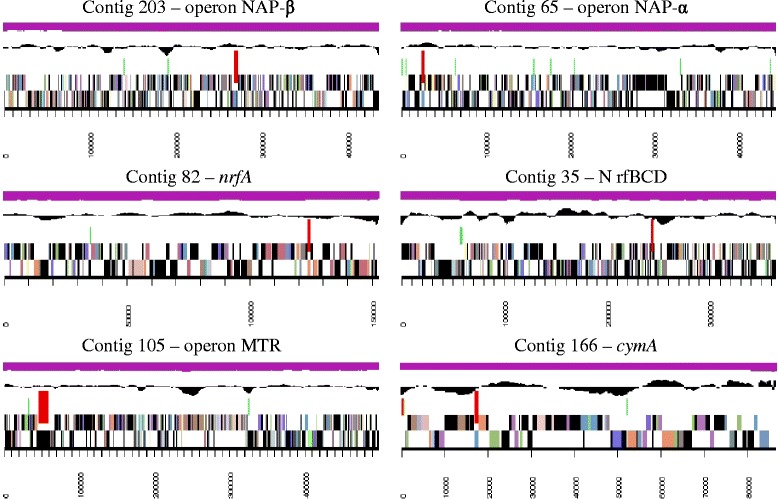
Graphical map of 6 contigs containing ORF involved in nitrate, nitrite and metal oxides utilization. Nitrate reduction (contig #203 and #65), dissimilative reduction of nitrite into ammonium, (contig #82 and #35) and metal reduction (contig #105 and #166). From bottom to the top: genes on forward strand (color by COG), genes on reverse strand (color by COG), operon/gene cited (pointed red), RNA genes (tRNAs green, rRNAs red, other RNAs black), GC content, GC skew

The assembly of non-contiguous finished draft genome consists of 43 contigs representing overall 4,879,425 pb. The DNA G + C content was 53.08 %. Using RAST and IMG/ER, 5770 and 5795 genes were respectively predicted. Among them 4149 and 5660 protein-coding genes and 108 and 135 RNAs were identified by RAST and IMG/ER, respectively. The properties and the statistics of the genome (IMG/ER data) are summarized in Table 3 and Fig. 3. Putative COG functions were assigned for 39.28 % of the protein-coding genes. The distribution of genes into COGs functional categories is presented in Table 4.

**Table 3:** Genome statistics of *S. algae* C6G3 (IMG/ER DOE-JGI)

Attribute	Value	% of Total^a^
Genome size (bp)	4,879,425	100.00
DNA coding (bp)	4,205,943	86.20
DNA G + C (bp)	2,589,944	53.08
DNA scaffolds	43	-
Total genes	5792	100
Protein coding genes	5660	97.72
RNA genes	132	2.28
Pseudo genes	0	0
Genes in internal clusters	4072	70.30
Genes with function prediction	4098	70.75
Genes assigned to COGs	2275	39.28
Genes with Pfam domains	4318	74.55
Genes with signal peptides	519	8.96
Genes with transmembrane helices	1268	21.89
CRISPR repeats	3	-

^a^The total is based on either the size of the genome in base pairs or the total number of protein coding genes in the annotated genome

**Table 4:** Number of genes associated with general COG functional categories

Code	Value	% age	Description
J	145	5.71	Translation, ribosomal structure and biogenesis
A	2	0.08	RNA processing and modification
K	190	7.49	Transcription
L	111	4.37	Replication, recombination and repair
B	0	0	Chromatin structure and dynamics
D	25	0.99	Cell cycle control, Cell division, chromosome partitioning
V	42	1.65	Defense mechanisms
T	176	6.93	Signal transduction mechanisms
M	142	5.59	Cell wall/membrane biogenesis
N	104	4.10	Cell motility
U	92	3.62	Intracellular trafficking and secretion
O	130	5.12	Posttranslational modification, protein turnover, chaperones
C	176	6.93	Energy production and conversion
G	85	3.35	Carbohydrate transport and metabolism
E	182	7.17	Amino acid transport and metabolism
F	60	2.36	Nucleotide transport and metabolism
H	129	5.08	Coenzyme transport and metabolism
I	90	3.55	Lipid transport and metabolism
P	136	5.36	Inorganic ion transport and metabolism
Q	43	1.69	Secondary metabolites biosynthesis, transport and catabolism
R	237	9.34	General function prediction only
S	241	9.50	Function unknown
-	3517	60.72	Not in COGs

The total is based on the total number of protein coding genes in the genome

### Insights into the genome sequence

Genome of *S. algae* C6G3 encodes genes for complete glycolysis and tricarboxylic acid (TCA) cycle. A focus has been made on the enzymes involved in the reductive respiratory reactions of the N-cycle (NAP, NRFA) and in extracellular electron transfer through the outer-membrane (Metal Transfer Reducing such as Fe(III) and Mn(III/IV)) on the basis of protein system described in *S. oneidensis* MR-1^T^.

### Nitrate reduction

**Fig. 4:**
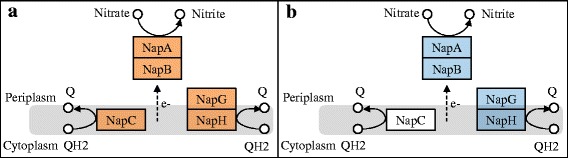
NAP complex functioning. The proposed electron-transfer pathway of periplasmic nitrate reductase and membrane bound electron donors of *S. algae* C6G3 (NAP-α-β) (**a**) and *S. oneidensis* MR-1^T^ (NAP-β) (**b**). Colored proteins are annotated from KEGG and putatively functional

Nitrate respiration involves two distinct enzyme systems: the NapAB localized in the periplasm and the membrane-bound nitrate reductase NarGHI enzyme localized on the cytoplasmic face of the cytoplasmic membrane. The sole nitrate reductase of *S. algae* C6G3 is NapAB (Fig. 4). As most of the *Shewanella* species, *S. algae* C6G3 genome encodes the two NAP isoforms, each comprising three catalytic subunits: NapA where nitrate reduction takes place, a di-haem cytochrome NapB and a maturation chaperone NapD. The two isoforms present different membrane-intrinsic subunits [34] named NAP-α (NapEDABC) and NAP-β (NapDAGHB). NAP-β (NapDAGBH) possesses NapGH, an iron–sulfur cluster ferredoxins instead of NapC. The functional differences between these systems may be explained by differential regulation in the composition of the available quinol pool. *S. oneidensis* MR-1^T^ encodes only Nap-β isoform in which NapC is lacking (Fig. 4). This membrane-anchored tetrahem *c*-Cyt mediates electron transport from the quinol pool to NapB. The function of NapC in NAP-β in *S. oneidensis* MR-1^T^ may be met by CymA, an homologue of periplasmic tetrahem *c*-Cyt of the NapC/NirT family, which is also found in *S. algae* C6G3 genome.

### Dissimilative reduction of nitrite into ammonium

**Fig. 5:**
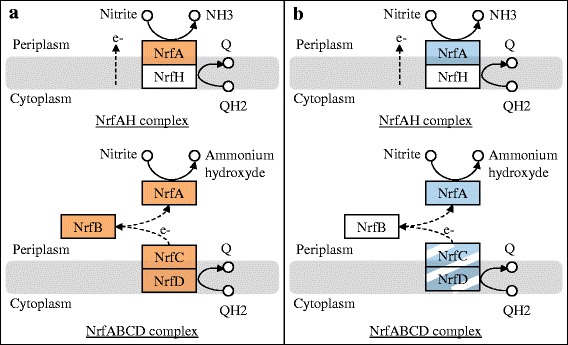
NRF complex functioning. The proposed electron-transfer pathway of dissimilatory nitrite reduction to ammonium of *S. algae* C6G3 (**a**) and *S. oneidensis* MR-1^T^ (**b**). Colored proteins are annotated from KEGG and putatively functional. Streaked genes are annotated but probably not functional

Nitrite can be reduced to ammonium (NH_4_) by a periplasmic nitrite reduction system (NRF) [35]. As NAP systems, there are two types of NRF: NrfABCD and NrfAH types. NrfA is the terminal reductase while NrfBCD/NrfH are responsible for electron transfer from menaquinol pool to NrfA. *Shewanella* strains are known for encoding NrfABCD system only. Genome annotations of *S. algae* C6G3 and *S. oneidensis* MR-1^T^ identify *nrfA* in both cases but *nrfBCD* were found in *S. algae* C6G3 only (Fig. 5). As previously described for NAP system, *S. oneidensis* MR-1^T^ genome lacks genes encoding for the specific compounds that deliver electrons to the terminal reductase (*nrfBCD)*. Indeed, *nrfB* is lacking and *nrfCD* are present but proposed to be pseudogene because of truncation [36]. Gao et al.*,* [37] suggested that the tetraheme *c*-Cyt CymA, a cytoplasmic membrane electron transport protein, is likely to be the functional replacement of both NapC and NrfBCD/NrfH allowing to NrfAH-like system to be efficient in *S. oneidensis* MR-1^T^.

### Metal oxide reduction

**Fig. 6:**
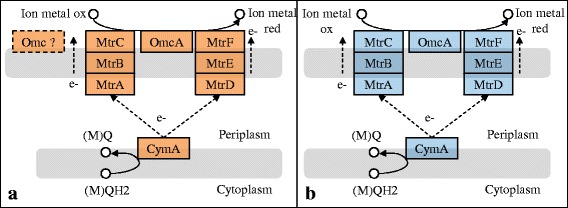
MTR pathway functioning. The proposed MTR extracellular electron-transfer pathway of *S. algae* C6G3 (**a**) and *S. oneidensis* MR-1^T^ (**b**). Colored proteins are annotated from KEGG and putatively functional

*S. algae* C6G3 can utilize extracellular mineral metal oxides of Fe(III) and Mn(III/IV) as respiratory electron acceptors (unpublished data). Inspection of its genome confirmed the presence of genes involved in pathway (i.e. metal-reducing or MTR pathway) for transferring electrons from the inner membrane through the periplasm and across the outer membrane where metal oxides are reduced [38]. In *S. algae* C6G3 genome, genes that encode MtrCBA and OmcA are located in the same region, which also includes *mtrD* (an *mtrA* homologue), *mtrE* (an *mtrB* homologue) and *mtrF* (an *mtrC* homologue) (Fig. 6).

Table 5 shows the BLASTP for amino acid sequences of MTR pathway in *S. algae* C6G3 versus those in metal-reducing *S. oneidensis* MR-1^T^*.*

**Table 5:** BlastP of MTR gene of *S. algae* C6G3 against MTR gene of *S. oneidensis* MR-1

MTR gene	% identities	E-value
MtrD	75	2e-172
MtrE	50	4e-125
MtrF	65	0.0
OmcA	65	0.0
OmcB/MtrC	51	3e-142
MtrA	86	0.0
MtrB	72	0.0

The numbers of genes found in the MTR clusters of the analyzed *Shewanella* strains varies from four, such as *omcA1-mtrC-mtrA-mtrB* in *Shewanella frigidimarina* [11], to nine, such as *mtrD-mtrE-mtrF-omcA1-undB-omcA1-mtrC-mtrA-mtrB* in *Shewanella halifaxensis* [39, 40]. CymA identified as the entry point for electrons into the MTR pathway [41] is not located in the MTR gene cluster in *S. algae* C6G3 as described in *S. oneidensis* MR-1^T^. Furthermore, *S. algae* C6G3 has an additional protein encoding for decahem *c*-Cyt of the OmcA/MtrC family. The role of this cytochrome is not defined. On RAST platform, this ORF has been annotated as mtrH in *S. algae* C6G3 and *S. halifaxensis* HAW-EB4^T^.

## Conclusion

The *Shewanella* genus comprises a diverse group of facultative anaerobes. Their ability to couple the oxidation of various carbon sources to the reduction of a broad range of terminal electron acceptors imparts a respiratory flexibility that allows colonization of varied and changeable marine and freshwater environments [39, 42, 43]. The occurrence of the two different NAP operons, NRF, and that of CymA in *S. algae* C6G3 accords with the renowned anaerobic respiratory flexibility of *Shewanella**.**S. algae* C6G3 is also capable of using solid Fe(III) and Mn(III/IV) as terminal electron acceptors. Reduction of these particulates occurs at the cell surface and is catalyzed by multihaem cytochromes whose properties are beginning to emerge.

## Additional files

Additional file 1:
**Table S1.** Presentation of positives carbon sources (Biolog GN2 microplateTM) & electron acceptors for S. algae strain C6G3 and ATCC 51192T and S. oneidensis MR-1T (differences are distinct in bold type). **Table S2.** Main fatty acids composition (90.6 %) of S. algae C6G3 and percentage of this fatty acids in S. algae ATCC 51192T (73.6 % of total pattern) and S. oneidensis MR-1T (92.5 % of total pattern). Additional file 2:
**Associated MIGS Record.**

## Abbreviation

TMAO: Trimethylamine N-oxide
