# Supplementary material for: Draft-genome sequence of Shewanella algae strain C6G3
Source: Stand Genomic Sci. 2015 Jul 23;10:43. doi: 10.1186/s40793-015-0022-0 (PMC4572631; doi:10.1186/s40793-015-0022-0)
Supplement: Additional file 1: — Table S1. Presentation of positives carbon sources (Biolog GN2 microplateTM) & electron acceptors for S. algae strain C6G3 and ATCC 51192T and S. oneidensis MR-1T (differences are distinct in bold type). Table S2. Main fatty acids composition (90.6 %) of S. algae C6G3 and percentage of this fatty acids in S. algae ATCC 51192T (73.6 % of total pattern) and S. oneidensis MR-1T (92.5 % of total pattern). [file 40793_2015_22_MOESM1_ESM.docx]

**Additional file 1: Table S1.** Presentation of positives carbon sources (Biolog GN2 microplate^TM^) & electron acceptors for *S. algae* strain C6G3 and ATCC 51192^T^ and *S. oneidensis* MR-1^T^ (differences are distinct in bold type).

| **Carbon sources** | [***Shewanella algae***](http://dx.doi.org/10.1601/nm.2916) **C6G3** | [***Shewanella algae***](http://dx.doi.org/10.1601/nm.2916) **ATCC 51192^T^** | [***Shewanella oneidensis***](http://dx.doi.org/10.1601/nm.2931) **MR-1^T^** |
| --- | --- | --- | --- |
| Carbohydrates (30) | Dextrin, Glycogen, **N-acetyl-D-Galactosamine**, N-acetyl-D-Glucosamine, L-Arabinose | Dextrin, Glycogen, N-acetyl-D-Glucosamine, L-Arabinose, **α-D-Glucose** | N-acetyl-D-Glucosamine, L-Arabinose |
| Organic acids (29) | Pyruvic Acid Methyl Ester, **Succinic Acid Mono-Methyl Ester**, **Acetic Acid, Formic Acid**, **α-Hydrobutyric Acid**, **β-Hydrobutyric Acid**, **α-Ketobutyric Acid**, **α-Ketoglutaric Acid**, **α-Ketovaleric Acid**, D,L-Lactic Acid, **Propionic Acid, Succinic Acid**, Bromosuccinic Acid, Succinamic Acid | | Pyruvic Acid Methyl Ester**, D-galactonic Acid Lactone**, **D-Glucuronic Acid**, D,L-Lactic Acid, Bromosuccinic Acid, Succinamic Acid |
| Amino acids (19) | L-Alanine, L-Alanyl-Glycine, **L-Glutamic Acid**, Glycyl-L-Aspartic Acid, Glycyl-L-Glutamic Acid, L-Leucine, L-Proline, L-Threonine | **D-alanine**, L-Alanine, L-Alanyl-Glycine, Glycyl-L-Aspartic Acid, Glycyl-L-Glutamic Acid, L-Leucine, **L-Ornithine**, **L-Phenylalanine**, L-Proline, **D-Serine**, **L-Serine**, L-Threonine | D-alanine, L-Alanine, L-Alanyl-Glycine, Glycyl-L-Aspartic Acid, Glycyl-L-Glutamic Acid |
| Alcohol (3) | - | - | - |
| Others (14) | Tween40, Tween80, Inosine, Uridine, Thymidine | Tween40, Tween80, **L-Alaninamide**, Inosine, Uridine, Thymidine, **Putrescine** | Tween80, **L-Alaninamide**, Inosine |
| Sources | Muddy intertidal sediments of Arcachon Bay | Red alga, *Jania sp.* | Lake Oneida |
| Electrons acceptors | O_2_, nitrite, Fe(III), Mn(III/IV), TMAO, **dimethylamine sulfoxide**, thiosulfate and **elementar sulfur**. | O_2_, nitrite, Fe(III), Mn(III/IV), U(VI) (strain BrY) [[1](#_ENREF_1)], TMAO, thiosulfate, **fumarate**. | O_2_, nitrite, Fe(III), Mn(III/VI), Cr(VI), U(VI), TMAO, thiosulfate, dimethylamine sulfoxide, **fumarate**, **sulfite**, sulfate and elementar sulfur |

**Additional file 1: Table S2.** Main fatty acids composition (90.6%) of [*S. algae*](http://dx.doi.org/10.1601/nm.2916) C6G3 and percentage of this fatty acids in [*S. algae*](http://dx.doi.org/10.1601/nm.2916) ATCC 51192^T^ (73.6% of total pattern) and [*S. oneidensis*](http://dx.doi.org/10.1601/nm.2931) MR-1^T^ (92.5% of total pattern).

| **Fatty acids** | [***S. algae***](http://dx.doi.org/10.1601/nm.2916) **C6G3** | [***S. algae***](http://dx.doi.org/10.1601/nm.2916) **ATCC 51192^T^** | [***S. oneidensis***](http://dx.doi.org/10.1601/nm.2931) **MR-1^T^** |
| --- | --- | --- | --- |
| < C15 | 16.0% | 8.5% | 8.5% |
| ≥ C15 | 84% | 91.5% | 91.5% |
| C12:0 | 4.6% | 0% | 0.4% |
| 3OH-C12:0 | **7.7%** | 0.5% | 3.1% |
| C14:0 | 2.8% | 0.6% | 3.8% |
| C15:0 br | 2.3% | **27.4%** | **20.4%** |
| C16:1ω7 | **35.2%** | **22.8%** | **31.2%** |
| C16:0 | **34.6%** | **19.9%** | **33.2%** |
| C18:1 ω9 | 3.4% | 2.4% | 0.4% |
