# Supplementary material for: Draft-genome sequence of Shewanella algae strain C6G3
Source: Stand Genomic Sci. 2015 Jul 23;10:43. doi: 10.1186/s40793-015-0022-0 (PMC4572631; doi:10.1186/s40793-015-0022-0)
Supplement: Additional file 2: — Associated MIGS Record. [file 40793_2015_22_MOESM2_ESM.docx]

# Associated MIGS Record

**Table S1.** Associated MIGS record

| **MIGS-ID** | field name | description |
| --- | --- | --- |
| **MIGS-1** | Submit to INSDC/Trace archives | [JPMA00000000](http://eutils.ncbi.nlm.nih.gov/entrez/eutils/efetch.fcgi?db=nucleotide&id=JPMA00000000&rettype=fasta&retmode=xml) |
| **1.1** | PID |  |
| **1.2** | Trace Archive |  |
| **MIGS-2** | MIGS CHECK LIST TYPE |  |
| **MIGS-3** | Project Name | IZOFLUX |
| **MIGS-4** | Geographic Location | Arcachon Bay, France |
| **4.1** | Latitude | N44° 40’ |
| **4.2** | Longitude | W1° 10’ |
| **4.3** | Depth | Top 10 cm of sediment |
| **4.4** | Altitude | Sea level |
| **MIGS-5** | Time of Sample collection | October 2007 |
| **MIGS-6** | Habitat (EnvO) | Muddy interdidal sediment |
| **6.1** | temperature | From 1 to 30°C |
| **6.2** | pH | 6–9; 8 |
| **6.3** | salinity | From 22 to 32 |
| **6.4** | chlorophyll | - |
| **6.5** | conductivity |  |
|  |  |  |
| **6.6** | light intensity |  |
| **6.7** | dissolved organic carbon (DOC) |  |
| **6.8** | current |  |
| **6.9** | atmospheric data |  |
| **6.10** | density |  |
| **6.11** | alkalinity |  |
| **6.12** | dissolved oxygen |  |
| **6.13** | particulate organic carbon (POC) |  |
| **6.14** | phosphate |  |
| **6.15** | nitrate |  |
| **6.16** | sulfates |  |
| **6.17** | sulfides |  |
| **6.18** | primary production |  |
| **MIGS-7** | Subspecific genetic lineage | Strain C6G3 |
| **MIGS-9** | Number of replicons | 1 |
| **MIGS-10** | Extrachromosomal elements | 0 |
| **MIGS-11** | Estimated Size | 4,879,425 |
| **MIGS-12** | Reference for biomaterial or Genome report | MS : 1999936125136254 |
| **MIGS-13** | Source material identifiers | SAMN03282794 |
| **MIGS-14** | Known Pathogenicity | Biosafety level 1 for ATCC 51192^T^ |
|  |  |  |
| **MIGS-15** | Biotic Relationship | Free living |
| **MIGS-16** | Specific Host | No host |
| **MIGS-17** | Host specificity or range (taxid) | - |
| **MIGS-18** | Health status of Host | - |
| **MIGS-19** | Trophic Level | Heterotroph |
| **MIGS-22** | Relationship to Oxygen | Facultative anaerobe |
| **MIGS-23** | Isolation and Growth conditions |  |
| **MIGS-27** | Nucleic acid preparation |  |
| **MIGS-28** | Library construction | Fragments (mean 200bp) |
| **28.1** | Library size | 200 pb |
| **28.2** | Number of reads | 1,444,981 |
| **28.3** | vector |  |
| **MIGS-29** | Sequencing method | Semiconductor Ion Torrent PGM |
| **MIGS-30** | Assembly |  |
| **30.1** | Assembly method | SeqMan NGen® (DNASTAR) |
| **30.2** | estimated error rate |  |
| **30.3** | method of calculation |  |
| **MIGS-31** | Finishing strategy | Non-contiguous finished |
| **31.1** | Status |  |
| **31.2** | coverage | 50× |
| **31.3** | contigs | 43 |
| **MIGS-32** | Relevant SOPs |  |
| **MIGS-33** | Relevant e-resources |  |
